# Comparative Analyses Between Vulnerability Biomarkers of Aging and Health Biomarkers in Middle-Aged and Older Female Adults

**DOI:** 10.3390/healthcare13060667

**Published:** 2025-03-18

**Authors:** Klara Karin Brigitte Knoblauch, Luana Froes Losnack, Gustavo Castillo Zacarias, Gabriel Gasparini Satyro, Rodrigo Villar, Anderson Saranz Zago

**Affiliations:** 1 Graduate Program in Movement Science, Sao Paulo State University (UNESP), São Paulo 17033-360, Brazil; k.knoblauch@tu-braunschweig.de (K.K.B.K.); luana.losnack@unesp.br (L.F.L.); gustavo.zacarias@unesp.br (G.C.Z.); 2 Center for Noncommunicable Diseases, Aging and Exercise Studies (CEDEE), Department of Physical Education, School of Sciences, Sao Paulo State University (UNESP), Bauru 17033-360, Brazil; g.satyro@unesp.br; 3 Cardiorespiratory & Physiology of Exercise Research Laboratory, Faculty of Kinesiology and Recreation Management, University of Manitoba, Winnipeg, MB R3T2N2, Canada; rodrigo.villar@umanitoba.ca

**Keywords:** vulnerability, biomarker, GDF-15, frailty, functional fitness, older age

## Abstract

Biological aging is normally associated with greater physiological changes which predispose individuals to adverse outcomes. In this way, the evaluation of vulnerability biomarkers and their relationships with other health biomarkers could contribute to the promotion of interventions and the improvement of older adults’ quality of life. Thereby, this study aimed to compare vulnerability biomarkers (Growth Differentiation Factor 15 (GDF-15), General Functional Fitness Index (GFFI), and frailty phenotype) and their influence on health markers (blood biochemistry, body composition, and hemodynamic variables) in middle-aged and older female adults. **Methods:** A cross-sectional observational study was conducted with community-dwelling females aged 54–84 with at least 6 months of experience with physical training. The participants were categorized based on functional fitness, frailty phenotype, and GDF-15 quartiles. The General Functional Fitness Index (GFFI) was assessed using the AAHPERD test battery, while frailty phenotype was determined using Fried’s criteria. GDF-15 levels were measured through ELISA. **Results:** A higher training status (TS) showed better functional fitness and favorable biochemical profiles, including lower total cholesterol (*p* = 0.006, η^2^p = 0.253), LDL cholesterol (*p* = 0.001, η^2^p = 0.346), triglycerides (*p* = 0.048, η^2^p = 0.195), and systolic blood pressure (*p* = 0.001, η^2^p = 0.333). Individuals classified as robust (non-frail) had better physical performance and lower total cholesterol (*p* = 0.002, η^2^p = 0.306) and LDL cholesterol (*p* = 0.014, η^2^p = 0.216) compared to those classified as frail and pre-frail. The GDF-15 quartile did not present differences in health markers between groups. **Conclusions:** These findings suggest that GFFI may be considered a health biomarker for middle-aged and female older adults while highlighting the need for further research on the role of biomarkers of vulnerability and healthy aging.

## 1. Introduction

As global populations age, it becomes increasingly important to understand older adults’ biological and functional vulnerabilities. Aging is accompanied by physiological changes that increase susceptibility to stressors such as infections, injuries, or chronic conditions [1].

Biological aging leads to a decline in the homeostatic and regenerative capacities of cells and tissues, aggravated by chronic inflammation, oxidative stress, and mitochondrial dysfunction [2,3,4]. These processes increase the risk of frailty—a clinical syndrome characterized by diminished physiological reserves, reduced mobility, and an increased risk of adverse health outcomes, such as falls, hospitalization, and mortality [5]. Therefore, biological aging is normally associated with greater vulnerability, a multifaceted concept including physical, cognitive, and social dimensions, predisposing individuals to adverse outcomes [6]. In this way, the comparison between vulnerability biomarkers, general functional fitness, frailty, and health markers in middle-aged and older female adults can help to identify the physiological markers that can best predict health risks and functional decline in this population, which could contribute to the promotion of interventions to improve quality of life.

The Growth Differentiation Factor 15 (GDF-15), a stress-responsive cytokine from the transforming growth factor-beta (TGF-β) superfamily [7,8], has gained attention as a vulnerability biomarker in aging research. Its expression increases in response to inflammation, oxidative damage, and mitochondrial dysfunction [9,10]—hallmarks of aging. Elevated GDF-15 levels have been linked to frailty, functional decline, and mortality. Additionally, GDF-15 is implicated in sarcopenia, which is characterized by the loss of skeletal muscle mass and strength, a major determinant of frailty [11,12]. It also plays a role in modulating appetite and energy homeostasis [13], suggesting its involvement in malnutrition among frail older adults. Importantly, GDF-15 levels are modifiable through interventions targeting inflammation, oxidative stress [14], and lifestyle factors, making it a promising tool for monitoring therapeutic effectiveness.

Furthermore, aging is generally associated with a decline in overall functional fitness [15], which includes strength, endurance, balance, and flexibility—essential components for maintaining independence and quality of life. Thereby, the assessment of General Functional Fitness Index (GFFI) levels can also be considered as a functional marker of vulnerability. Assessing GFFI provides a comprehensive functional assessment of overall fitness and health, crucial for maintaining independence and reducing risks of falls and disability, and enables targeted interventions to enhance resilience.

Another condition of vulnerability commonly associated with aging is frailty [16]. It reflects a decline in physiological reserves across multiple systems, increasing susceptibility to stressors and adverse outcomes such as disability and mortality [17]. Chronic inflammation, driven by elevated cytokines such as interleukin-6 (IL-6) and tumor necrosis factor-alpha (TNF-α), underpins frailty progression [18]. Frailty is clinically assessed using instruments like the Fried frailty phenotype [6], which evaluates unintentional weight loss, weakness, slowness, exhaustion, and low physical activity.

Addressing vulnerability through biomarkers, clinical assessments, and fitness measures is a critical priority in aging research. By comparing GDF-15, functional fitness, and frailty with blood biochemistry, body composition, and blood pressure offers opportunities to develop personalized assessments, and intervention strategies that enhance resilience, promote healthy aging, and reduce age-related morbidity. However, this topic is still poorly understood and requires further research. Additionally, although GDF-15 is a biological biomarker, it is believed that both GFFI and the frailty assessment can also be considered important health markers that should be considered. 

Thereby, the purpose of this study was to compare vulnerability biomarkers (GDF-15, GFFI, and frailty phenotype) with blood biochemistry, body composition, and blood pressure in middle-aged and older female adults. The knowledge gained from these findings can contribute to more effective health assessments, better management of aging-related conditions in clinical practice, and the development of targeted interventions to enhance the quality of life in this population.

## 2. Materials and Methods

A cross-sectional observational study was conducted at the “Center for the Study of Noncommunicable Diseases, Aging and Physical Exercise (CEDEE)” of the School of Sciences of the Sao Paulo State University (UNESP), Bauru, São Paulo, Brazil. Before the evaluations, the participants signed an informed consent form after the principal investigator clarified all their questions and concerns. All procedures were approved by the Research Ethics Committee of the Faculty of Sciences/UNESP-Bauru (protocol no.: 323.427), which is in accordance with the Declaration of Helsinki.

Data collection was performed on thirty-eight community-dwelling females aged 54–84 who had at least 6 months of experience in physical exercise. To be included in the study, participants had to meet the following criteria: postmenopausal females; nonsmokers; no medical, cardiological, orthopedic, and/or musculoskeletal restrictions that could compromise test evaluation; alcoholic beverage consumption of up to two drinks daily; and no use of any known medication that affects glucose metabolism or renal hemodynamics. Older adults diagnosed with dementia, psychiatric disorders, cognitive impairment, and stroke were excluded from this study. This information was collected during participants’ interviews, where a standard screening health questionnaire was used to check for the eligibility criteria as described above. Additionally, participants who did not complete all assessments were excluded from the data analysis. In fact, 57 participants were selected in the first screening, but 19 participants were excluded from the sample according to the aforementioned inclusion criteria (n = 38 participants).

This study was conducted according to the “Strengthening the Reporting of Observational Studies in Epidemiology” (STROBE) Statement guidelines (http://www.equatornetwork.org/). To test the study hypotheses, participants were divided into three different classifications: (a) frail, pre-frail, and robust, based on Fried’s phenotype criteria (weight loss, exhaustion, low physical activity, slow gait, and weak grip strength). The cut-off points were adjusted according to Boreskie [19], which took into account the classification of community-dwelling middle-aged and older female adults; (b) weak, regular, and good training status (TS) levels, assessed by the AAHPERD test battery (American Alliance for Health, Physical Education, Recreation and Dance), assessing agility, coordination, flexibility, strength, and aerobic endurance; and (c) GDF-15 quartile, obtained through the quantification of GDF-15 by ELISA.

### 2.1. Measurements

General Functional Fitness Index (GFFI) assessments: The battery of functional fitness assessments proposed by the AAHPERD was used to determine the participants’ training status (TS). The battery consisted of five standardized motor tests conducted in the following order: flexibility, coordination, muscular strength and upper arm muscular endurance, agility and dynamic balance, and endurance, as previously described [20,21,22]. For greater reliability of the results, the endurance test was performed on a flat athletic track. Participants were classified according to normative values [20,22] based on the sum of scores obtained in each test to calculate the GFFI. The total score possible was 500 points, where each test had a maximum value of 100 points. The GFFI values were classified as weak TS (<200 points), regular TS (200–300 points), and good TS (>300 points). The AAHPERD battery was selected for its strong reliability and validity in the older adult population with test–retest reliability coefficients ranging from 0.80 to 0.99 for each component [23].

Frailty phenotype: All participants underwent frailty phenotype assessments that consisted of five criteria, as follows: (a) unintentional weight loss (~4.5 kg) in the last year; (b) CES-D Depression Scale; (c) level of physical activity, assessed through the Paffenbarger questionnaire; (d) walking speed (4.57 m); and (e) handgrip strength, as previously described in Fried et al. [6]. Scores were assigned for each criteria, allowing for the classification of the frailty phenotype as robust (score of 0), pre-frail (score of 1 to 2), and frail (score of 3 to 5). As aforementioned, Boreskie’s cut-off points were used to determine the frailty phenotype classification because they are based on community-dwelling middle-aged and older adults [19].

Body composition assessments: For body composition, a dual-energy X-ray absorptiometry method (DXA) was performed (Discovery, Wi-HOLOGIC INC device, Bedford, MA, USA) to determine body composition (lean mass, fat mass, bone mineral content, and sarcopenic index). Briefly, the equipment generated X-rays at two different energies and used the differential attenuation of the X-ray beam. These two energies were used to calculate the tissue composition in the scanned area [24,25]. Data were analyzed by Hologic APEX software (version 13.2.5). Body mass index (BMI) was also calculated by dividing the weight by the height squared (kg/m^2^).

Arterial blood pressure: Systolic blood pressure (SBP) and diastolic blood pressure (DBP) were measured according to the Brazilian Hypertension Guidelines [26] by the auscultatory method using an aneroid sphygmomanometer (Welch Allyn DS44, Wan Med^®^, St. Skaneateles Falls, NY, USA) and a stethoscope (3MTM, Classic III, Littmann^®^, St. Paul, MN, USA) positioned on the brachial artery. Three measurements were performed on different days [26].

Blood biochemistry: Blood samples were collected between 07:00 AM and 08:30 AM after a 12 h fasting period. Participants were instructed to refrain from exercise on the day of testing. Participants rested for at least 10 min while sitting in a chair. A total of 10 mL of blood was then drawn by a certified phlebotomist using disposable syringes and needles. Blood samples were immediately transferred to dry tubes for further analysis. Blood samples were centrifuged at 4000 rpm for 10 min for serum separation. Serum samples were used to determine total cholesterol (TC), high-density lipoprotein cholesterol (HDL-C), low-density lipoprotein cholesterol (LDL-C), triglycerides (TG), and glycemia (GL) by the spectrophotometry method using a biochemical analyzer with specific reagents (A-15, BioSystems SA, Barcelona, CAT, Spain).

GDF-15 circulating levels: To quantify GDF-15 concentrations in serum samples, the Elabscience^®^ Human GDF-15 ELISA (Enzyme-Linked Immunosorbent Assay) Kit, a sandwich ELISA assay, was used (Catalog No: E-EL-H0080, Elabscience Biotechnology Co., Ltd., Houston, Texas, USA). The protocol followed the manufacturer’s instructions. All samples were run in duplicate.

### 2.2. Statistical Analysis

The Shapiro–Wilk test assessed data normal distribution, and Levene’s test assessed the homogeneity of variances. Quantitative variables were analyzed using ANCOVA (Analysis of Covariance), adjusting for BMI and age, with *p* < 0.05 indicating statistical significance. Each variable was analyzed separately, with GFFI, frailty phenotype, and GDF-15 quartiles as independent variables. Tukey’s post hoc test was used to identify the differences between groups. A 95% confidence interval (95% CI) was applied and data were expressed as mean and SD. Statistical analysis was performed using the Jamovi software (version 2.6) [27].

## 3. Results

**Figure 1:**
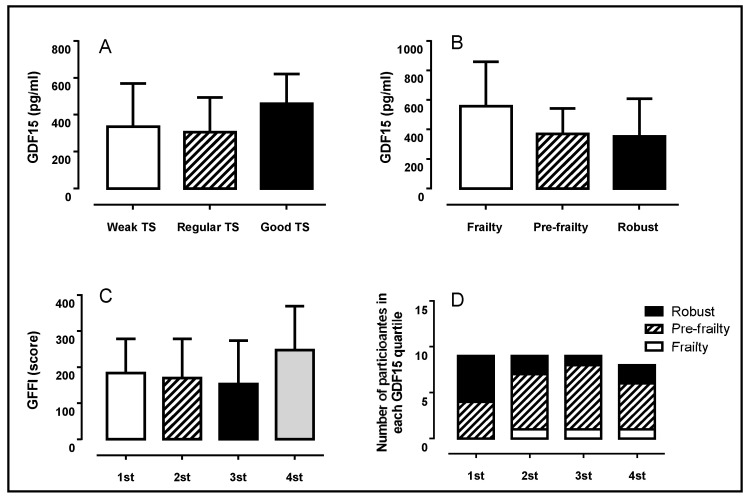
Growth Differentiation Factor 15 (GDF-15) according to different levels of training status—TS (**A**) and frailty classification (**B**). General Functional Fitness Index (GFFI) according to GDF-15 quartile (**C**) and number of participants in each quartile according to frailty classification (**D**).

A total of 38 participants (age = 66.8 ± 6.5/BMI = 28.8 ± 5.4) were included in this study. Figure 1 illustrates GDF-15 levels across training status (TS) and frailty classification, and the GFFI according to GDF-15 quartile, and the number of participants with frailty in each GDF-15 quartile. Although GDF-15 tended to have higher levels in participants with good TS (Panel A), a non-significant variation across different TS levels was observed (*p* = 0.347, η^2^p = 0.052). The same tendency was also observed in GDF-15 concentration for the participants classified as frail (Panel B), but non-significant differences were observed between participants classified as frail, pre-frail, or robust (*p* = 0.621, η^2^p = 0.069).

The General Functional Fitness Index (GFFI) demonstrated a tendency to be higher in the fourth quartile of GDF-15 (Panel C); however, it did not reach statistical significance (*p* = 0.325, η^2^p = 0.101). Participants in the higher GDF-15 quartiles (second, third, and fourth) were more likely to fall into the frail classification (Panel D), suggesting a potential link between GDF-15 levels and frailty. No participant with low GDF-15 levels was classified as frail.

Table 1 highlights the differences in physical performance, biochemical markers, and body composition across estimated TSs (weak, regular, and good). Significant improvements in physical performance metrics, including coordination (*p* = 0.001, η^2^p = 0.285), flexibility (*p* = 0.004, η^2^p = 0.261), muscular strength (*p* = 0.001, η^2^p = 0.613), agility and dynamic balance (*p* = 0.001, η^2^p = 0.335), and cardiovascular endurance (*p* = 0.001, η^2^p = 0.548), were observed with an increased TS. Participants with a good TS had the highest GFFI scores (371.7 ± 44) compared to those with a regular (224.1 ± 37) and weak TS (119.9 ± 42; *p* < 0.001, η^2^p = 0.861).

Total cholesterol (*p* = 0.006, η^2^p = 0.253), LDL cholesterol (*p* = 0.001, η^2^p = 0.346), and triglycerides (*p* = 0.048, η^2^p = 0.195) were significantly lower in participants with a good TS compared with a weak and regular TS. Notably, HDL cholesterol was significantly higher in the good TS group (61.6 ± 7 mg/dL; *p* = 0.002, η^2^p = 0.296). GDF-15 and glucose values did not show any statistical differences between TS groups.

Body composition and blood pressure showed non-significant differences between groups, except for systolic blood pressure, which was significantly lower in the good TS compared to the weak TS group (*p* = 0.001, η^2^p = 0.333). The sarcopenic index showed a trend toward lower values in participants with a good TS, although this difference was not statistically significant (*p* = 0.055, η^2^p = 0.153).

Differences in general functional fitness assessment, blood biochemistry, and body composition, and blood pressure according to frailty classification are shown in Table 2. Robust participants exhibited significantly higher muscular strength (27.8 ± 5 repetitions; *p* = 0.050, η^2^p = 0.321) and GFFI scores (262.7 ± 126; *p* = 0.009, η^2^p = 0.237) compared to the frail and pre-frail groups.

Regarding blood biochemistry, robust participants had significantly lower LDL cholesterol levels (101.0 ± 52 mg/dL; *p* = 0.014, η^2^p = 0.216) and total cholesterol levels (161.8 ± 46 mg/dL; *p* = 0.002, η^2^p = 0.306) compared to people with frail and pre-frail conditions. Other biochemical markers, including GDF-15 and glucose, showed non-significant trends across frailty classifications.

Body composition parameters and blood pressure did not reach significance across the frailty group classifications. Systolic blood pressure showed no statistical significance between groups (*p* = 0.284, η^2^p = 0.071) although high values were observed for the frail group (139.3 ± 14 mmHg).

Table 3 presents the analysis of the GDF-15 quartiles related to physical and biochemical measures, body composition, and blood pressure. GDF-15 levels increased significantly across quartiles, from the first quartile (163.4 ± 46 pg/mL) to the fourth quartile (686.8 ± 168 pg/mL; *p* = 0.001, η^2^p = 0.833). However, physical performance metrics such as coordination, flexibility, muscular strength, agility, and cardiovascular endurance did not demonstrate statistically significant differences across quartiles. Although participants in the fourth quartile exhibited the highest GFFI values (247.5 ± 122), no difference was found in the GFFI scores between groups (*p* = 0.325, η^2^p = 0.101).

Blood biochemistry, body composition, and blood pressure showed no statistical significance across quartiles (Table 3). 

## 4. Discussion

As previously mentioned, the purpose of this study was to compare vulnerability biomarkers (GDF-15, GFFI, and frailty phenotype) with blood biochemistry, body composition, and blood pressure in middle-aged and older female adults. Briefly, the results of this study indicated that individuals in the good TS group showed higher GFFI scores and HDL-cholesterol, and lower total cholesterol, LDL-cholesterol, triglycerides, and systolic blood pressure, highlighting the potential of GFFI to be used as a vulnerability functional marker to be incorporated into clinical practice for the better health management of older adults. Moreover, robust individuals showed higher GFFI scores and lower total cholesterol and LDL-cholesterol values than frail individuals, confirming frailty phenotype as a vulnerability biomarker as well.

Biomarkers like GDF-15, frailty, and GFFI levels are related and play crucial roles in assessing vulnerability in older adults. Elevated GDF-15 levels may signal early biological changes predisposing individuals to frailty and functional decline, while frailty and reduced fitness provoke systemic stress, potentially increasing GDF-15 levels. Understanding these dynamics is essential for identifying at-risk populations and designing effective interventions. However, these expected relationships were not revealed by our results. No differences were demonstrated in Figure 1A–C. It was expected that individuals with lower GFFI (weak TS) and those classified as frail would present high GDF-15 values, but these results were not confirmed.

Recently, GDF-15 has been strongly associated with aging and longevity [28,29,30]. The authors observed that, in healthy and young individuals, the circulating GDF-15 concentrations tended to remain lower. However, these concentrations increased in older adults, especially in the presence of pathological conditions [31,32]. Conversely, the literature indicates that the engagement of physical exercise can also stimulate the secretion of GDF-15. Kleinert et al. [33] observed a 34% increase in plasma GDF-15 levels after a single session of physical exercise. In addition, Zhang et al. [34] showed that the acute increase in GDF-15 induced by physical exercise was associated with improved energy metabolism in obese older adults. It is believed that the increase in GDF-15 levels may represent a compensatory metabolic response to mechanical and metabolic stress induced by physical exercise [31,35], aiding in muscle repair and regeneration after acute exercise [36]. However, it is important to highlight that these results were observed after a single acute session of physical exercise. In the current study, the training status (chronic effect) of older adults was evaluated (6 months of experience with exercise training).

Thereby, the chronic effect of exercise on GDF-15, GFFI, and frailty status still requires further study, especially if we take into consideration that both GFFI and the frailty status reflect the chronic condition of the individual. In the literature, the chronic effects of exercise on GDF-15 concentrations remain inconclusive, since some studies have shown an increase [34], a decrease [37], or no effect [38]. As an example, Torrens-Mas et al. and Tavenier et al. [39,40] demonstrated the potential role of GDF-15 as a biomarker of aging, since high concentrations of GDF-15 were associated with a lower nutritional status, lower functional performance, frailty, and high values of inflammatory and glycemic markers. Conversely, Seo et al. [37] did not report differences in GDF-15 concentration after 16 weeks of resistance training in older adults. Although GDF-15 has been considered a marker of vulnerability in older adults, further studies are still needed to reach a consensus.

Overall, our results showed that the higher TS group demonstrated better functional fitness and more favorable biochemical and hemodynamic profiles than the weak TS group (Table 1). Regarding frailty status, robust individuals showed higher GFFI scores and improvements in some biochemical profile variables than pre-frail and frail individuals (Table 2). GDF-15 levels did not reach statistical significance for any variable across the quartile (Table 3). Interestingly, participants in the 4th GDF-15 quartile tended to exhibit increased GFFI score and a larger presence of frailty and pre-frail individuals (Figure 1 C-D), which partially supports previous findings that link elevated GDF-15 levels with adverse health outcomes in aging populations [9,10]. However, the lack of significance in this study may reflect the relatively small sample size or the inclusion of physically active older adults (chronic effect of exercise), which could have attenuated the expected relationships.

Participants with a better TS (Table 1) exhibited more favorable biochemical profiles, including lower LDL cholesterol and triglycerides, higher HDL cholesterol levels, and lower systolic blood pressure values, reinforcing the role of exercise in promoting metabolic health [41]. A good TS was also significantly related to improved physical performance metrics, such as coordination, flexibility, muscular strength, agility, and cardiovascular endurance. Studies developed by our group have also found similar results; for example, Da Silva et al. [42] reported that total cholesterol, HDL-C, TG, GL, body mass index, body fat percentage, and systolic and diastolic blood pressure values were better in participants with higher GFFI scores (>300 points). These findings align with previous studies highlighting the role of physical fitness in mitigating aging-related vulnerabilities [11,15].

Frailty classification revealed significant differences in physical performance and biochemical markers (Table 2). Participants classified as robust exhibited higher GFFI scores and lower LDL and total cholesterol levels compared to their frail counterparts. These results are consistent with the existing literature, which identifies frailty as a multidimensional construct associated with reduced physiological reserves and increased susceptibility to adverse health outcomes [6,17].

This study contributes to the growing body of evidence supporting the integration of biomarkers and health markers for middle-aged and older female adults, such as GDF-15, frailty classification, and functional fitness assessments, into clinical practice. While GDF-15 has been extensively studied as a marker of inflammation, oxidative stress, and mitochondrial dysfunction [2,4], its potential as a predictor of health markers requires further exploration. However, frailty phenotype assessment, and especially the GFFI score, have shown to be good markers for the management of health in middle-aged and older female adults.

Future studies should address the limitations of the present study, including its cross-sectional design and relatively small sample size. Longitudinal studies are needed to establish causal relationships between GDF-15, frailty, functional fitness, and other health markers. Moreover, the potential modulatory effects of exercise and lifestyle interventions on the vulnerability biomarkers warrant further investigation, as these could inform personalized strategies to enhance resilience and reduce age-related morbidity. It is essential to consider lifestyle and biological markers to fully understand and address frailty in older adults. Exploring the influence of GDF-15, frailty phenotype, and GFFI score on specific health variables—such as blood pressure, body composition, and biochemistry—in larger, more diverse populations would also be valuable. Understanding the mechanisms underlying these relationships could provide new insights into the biological processes driving aging and vulnerability, though further investigation is needed.

## 5. Conclusions

Overall, individuals with higher training status and lower frailty levels demonstrated higher functional fitness scores and more favorable blood biochemical and hemodynamic profiles among middle-aged and older female adults, while highlighting the need for further research on the role of vulnerability biomarkers and healthy aging. Although GFFI is not yet widely recognized in the current geriatric research literature as a vulnerability marker in older adults, the results of the current study suggest that this index has great potential to be incorporated into clinical practice due to its favorable results on health parameters.

Although GDF-15 levels are also considered to be biological biomarkers of vulnerability, no differences were found between GDF-15 levels and any other variable in this study; However, further studies need to be carried out to confirm these results.

## Figures and Tables

**Table 1:** Estimated training status, blood biochemistry, body composition, and blood pressure values according to GFFI classification.

	Weak TS	Regular TS	Good TS	*p*	ƞ^2^p
**Estimated Training Status**					
Coordination (seconds)	20.3 ± 8.5	13.2 ± 2.2	10.5 ± 1.7 ^a^	0.001	0.285
Flexibility (cm)	48.6 ± 13.2	61.8 ± 8.5	63.3 ± 6.8 ^a^	0.004	0.261
Muscular strength (repetition)	21.0 ± 3.9	24.2 ± 3.5	32.1 ± 2.3 ^ab^	0.001	0.613
Agility and dynamic balance (seconds)	33.2 ± 9.8	23.5 ± 4.1 ^a^	20.7 ± 2.0 ^a^	0.001	0.335
Cardiovascular endurance (seconds)	655.3 ± 92	547.6 ± 25 ^a^	475.0 ± 27 ^a^	0.001	0.548
General Functional Fitness Index (score)	119.9 ± 42	224.1 ± 37 ^a^	371.7 ± 44 ^a^	0.000	0.861
**Blood Biochemistry**					
GDF-15 (pg·mL^−1^)	366.3 ± 234	306.5 ± 188	461.6 ± 160	0.347	0.052
Total cholesterol (mg/dL)	199.0 ± 40	219.0 ± 19	154.7 ± 37 ^ab^	0.006	0.253
HDL cholesterol (mg/dL)	44.3 ± 16	32.0 ± 10	61.6 ± 7 ^ab^	0.002	0.296
LDL cholesterol (mg/dL)	140.3 ± 44	166.0 ± 29	80.0 ± 32 ^ab^	0.001	0.346
VLDL cholesterol (mg/dL)	14.3 ± 11	20.9 ± 13	13.0 ± 2	0.353	0.058
Triglycerides (mg/dL)	95.6 ± 47	125.6 ± 45	65.3 ± 10 ^b^	0.048	0.195
Glucose (mg/dL)	115.8 ± 41	102.1 ± 8	95.2 ± 13	0.310	0.065
**Body Composition**					
Lean mass (kg)	41.0 ± 9	35.5 ± 4	34.0 ± 4	0.095	0.126
Lean mass (%)	57.4 ± 5	57.1 ± 4	55.0 ± 7	0.637	0.025
Fat mass (kg)	29.2 ± 9	25.0 ± 5	27.0 ± 9	0.540	0.035
Fat mass (%)	39.8 ± 5	39.8 ± 5	41.9 ± 7	0.706	0.020
Bone mineral content (kg)	1.9 ± 0.4	1.8 ± 0.2	1.8 ± 0.3	0.935	0.004
Sarcopenic index	6.8 ± 1.4	6.1 ± 0.7	5.6 ± 0.6	0.055	0.153
Body mass index (kg/m^2^)	29.9 ± 6	27.4 ± 3	26.3 ± 3	0.220	0.083
**Blood Pressure**					
Systolic blood pressure (mmHg)	133.2 ± 12	120.9 ± 10	113.5 ± 11 ^a^	0.001	0.333
Diastolic blood pressure (mmHg)	76.5 ± 8	80.0 ± 7	71.6 ± 8	0.194	0.092

TS—training status; η^2^p—ETA partial square. ^a^ *p* < 0.05 vs. weak TS; ^b^ *p* < 0.05 vs. regular TS.

**Table 2:** Estimated training status, blood biochemistry, body composition, and blood pressure values according to frailty classification.

	Frail	Pre-Frail	Robust	*p*	ƞ^2^p
**Estimated Training Status**					
Coordination (seconds)	24.5 ± 9	17.6 ± 8	14.3 ± 5	0.240	0.110
Flexibility (cm)	54.3 ± 18	50.7 ± 13	59.6 ± 11	0.249	0.092
Muscular strength (repetition)	16.3 ± 7	22.8 ± 4	27.8 ± 5 ^ab^	0.050	0.321
Agility and dynamic balance (seconds)	33.9 ± 8	30.6 ± 10	24.9 ± 5	0.266	0.093
Cardiovascular endurance (seconds)	577.0 ± 100	616.0 ± 107	564.0 ± 106	0.981	0.049
General Functional Fitness Index (score)	93.6 ± 50	163.6 ± 86	262.7 ± 126 ^ab^	0.009	0.237
**Blood Biochemistry**					
GDF-15 (pg·mL^−1^)	558.2 ± 302	370.5 ± 173	354.1 ± 256	0.621	0.069
Total cholesterol (mg/dL)	238.0 ± 44	203.1 ± 28	161.8 ± 46 ^ab^	0.002	0.306
HDL cholesterol (mg/dL)	36.9 ± 15	47.0 ± 17	46.5 ± 16	0.634	0.026
LDL cholesterol (mg/dL)	172.5 ± 42	142.3 ± 39	101.0 ± 52 ^ab^	0.014	0.216
VLDL cholesterol (mg/dL)	28.5 ± 22	13.8 ± 10	14.2 ± 7	0.078	0.135
Triglycerides (mg/dL)	142.6 ± 110	93.7 ± 31	77.4 ± 29	0.074	0.170
Glucose (mg/dL)	135.6 ± 61	105.4 ± 31	110.2 ± 34	0.376	0.054
**Body Composition**					
Lean mass (kg)	40.0 ± 6	37.3 ± 6	40.8 ± 12	0.525	0.036
Lean mass (%)	57.4 ± 5	56.7 ± 6	56.9 ± 5	0.984	0.001
Fat mass (kg)	27.4 ± 2	27.8 ± 9	28.7 ± 8	0.946	0.003
Fat mass (%)	39.6 ± 5	40.4 ± 6	40.1 ± 5	0.974	0.002
Bone mineral content (kg)	2.0 ± 0.4	1.8 ± 0.3	2.0 ± 0.3	0.230	0.081
Sarcopenic index	6.8 ± 0.9	6.4 ± 1.2	6.4 ± 1.6	0.928	0.004
Body mass index (kg/m^2^)	29.2 ± 1.7	28.4 ± 5.0	29.3 ± 6.7	0.883	0.007
**Blood Pressure**					
Systolic blood pressure (mmHg)	139.3 ± 14	126.8 ± 14	124.1 ± 14	0.284	0.071
Diastolic blood pressure (mmHg)	78.3 ± 5.5	77.8 ± 9.9	72.1 ± 6.0	0.181	0.096

ƞ^2^p—ETA partial square. ^a^ *p* < 0.05 vs. frail; ^b^ *p* < 0.05 vs. pre-frail.

**Table 3:** Estimated training status, blood biochemistry, body composition, and blood pressure values according to GDF-15 quartile.

	1st	2nd	3rd	4th	*p*	ƞ^2^p
**Estimated Training Status**						
Coordination (sec)	19.3 ± 12	16.2 ± 4	1935 ± 8	14.6 ± 4	0.439	0.066
Flexibility (cm)	56.0 ± 14	54.6 ± 13	44.9 ± 13	58.2 ± 12	0.219	0.141
Muscular strength (repetition)	24.4 ± 4	22.0 ± 6	21.3 ± 6	27.3 ± 4	0.121	0.170
Agility and dynamic balance (sec)	29.4 ± 9	29.1 ± 7	30.1 ± 10	27.4 ± 13	0.977	0.010
Cardiovascular endurance (sec)	640.9 ± 106	604.6 ± 116	573.5 ± 93	541.7 ± 118	0.401	0.119
GFFI (score)	183.6 ± 95	169.8 ± 109	153.4 ± 120	247.5 ± 122	0.325	0.101
**Blood Biochemistry**						
GDF-15 (pg·mL^−1^)	163.4 ± 46	288.8 ± 60 ^a^	422.4 ± 34 ^ab^	686.8 ± 168 ^abc^	0.001	0.833
Total cholesterol (mg/dL)	175.7 ± 41	202.4 ± 37	210.8 ± 35	177.2 ± 50	0.204	0.132
HDL cholesterol (mg/dL)	44.6 ± 14	46.4 ± 10	53.8 ± 22	43.7 ± 18	0.587	0.058
LDL cholesterol (mg/dL)	117.3 ± 48	142.2 ± 39	137.8 ± 45	120.2 ± 62	0.643	0.050
VLDL cholesterol (mg/dL)	13.8 ± 9	13.8 ± 9	19.1 ± 14	13.2 ± 9	0.654	0.049
Triglycerides (mg/dL)	89.1 ± 33	89.0 ± 35	107.8 ± 69	85.2 ± 34	0.779	0.042
Glucose (mg/dL)	111.7 ± 38	110.5 ± 49	112.2 ± 36	104.4 ± 17	0.968	0.008
**Body Composition**						
Lean mass (kg)	43.2 ± 14.0	35.8 ± 2.8	40.6 ± 6.8	35.2 ± 4.9	0.173	0.142
Lean mass (%)	56.4 ± 4	58.4 ± 7	56.2 ± 5	56.9 ± 7	0.882	0.020
Fat mass (kg)	31.2 ± 9	25.0 ± 9	30.0 ± 7	25.7 ± 8	0.349	0.096
Fat mass (%)	41.0 ± 4	38.6 ± 7	41.0 ± 5	39.9 ± 7	0.845	0.025
Bone mineral content (kg)	1.9 ± 0.4	1.7 ± 0.2	1.9 ± 0.4	1.9 ± 0.3	0.682	0.045
Sarcopenic index	7.1 ± 1.9	5.9 ± 0.5	6.8 ± 1.0	6.1 ± 1.3	0.186	0.138
Body mass index (kg/m^2^)	32.4 ± 7.8	25.9 ± 3.9	29.9 ± 3.2	27.0 ± 4.7	0.059	0.224
**Blood Pressure**						
Systolic blood pressure (mmHg)	124.7 ± 17	130.0 ± 11	132.9 ± 14	118.5 ± 13	0.201	0.137
Diastolic blood pressure (mmHg)	77.0 ± 8	75.9 ± 7	76.5 ± 10	72.1 ± 9	0.683	0.046

GFFI—General Functional Fitness Index, ƞ^2^p—ETA partial square. ^a^ *p* < 0.05 vs. first quartile; ^b^ *p* < 0.05 vs. second quartile; ^c^ *p* < 0.05 vs. third quartile.

## Data Availability

Data are contained within the article.

## Abbreviations

The following abbreviations are used in this manuscript:

GDF-15	Growth Differentiation Factor 15
TS	Training Status
GFFI	General Functional Fitness Index
AAHPERD	American Alliance for Health, Physical Education, Recreation and Dance
DXA	Dual-energy X-ray Absorptiometry
SBP	Systolic Blood Pressure
DBP	Diastolic Blood Pressure
TC	Total Cholesterol
HDL-C	High-Density Lipoprotein Cholesterol
LDL-C	Low-Density Lipoprotein Cholesterol
TG	Triglycerides
GL	Glucose
η^2^p	ETA Partial Square
